# Effects of Creatine Supplementation on the Myostatin Pathway and Myosin Heavy Chain Isoforms in Different Skeletal Muscles of Resistance-Trained Rats

**DOI:** 10.3390/nu15092224

**Published:** 2023-05-08

**Authors:** Marianna Rabelo de Carvalho, Ellen Fernandes Duarte, Maria Lua Marques Mendonça, Camila Souza de Morais, Gabriel Elias Ota, Jair José Gaspar-Junior, Wander Fernando de Oliveira Filiú, Felipe Cesar Damatto, Marina Politi Okoshi, Katashi Okoshi, Rodrigo Juliano Oliveira, Paula Felippe Martinez, Silvio Assis de Oliveira-Junior

**Affiliations:** 1 Graduate Program in Health and Development in the Midwestern Region, Federal University of Mato Grosso do Sul (UFMS), Campo Grande 79070-900, MS, Brazil; mariannarabelo5@gmail.com (M.R.d.C.); ellenduarte_12@hotmail.com (E.F.D.); marialuamarques@hotmail.com (M.L.M.M.); nutricionistacamila@outlook.com (C.S.d.M.); gabriel.elias.ota@gmail.com (G.E.O.); gasparjr.ft@gmail.com (J.J.G.-J.); rodrigo.oliveira@ufms.br (R.J.O.); paula.martinez@ufms.br (P.F.M.); 2 Faculty of Pharmaceutical Sciences, Food and Nutrition, Federal University of Mato Grosso do Sul (UFMS), Campo Grande 79070-900, MS, Brazil; wander.filiu@gmail.com; 3 Internal Medicine Department, Botucatu Medical School, Sao Paulo State University (UNESP), Botucatu 18618-687, SP, Brazil; felipedamatto@hotmail.com (F.C.D.); marina.okoshi@unesp.br (M.P.O.); katashi.okoshi@unesp.br (K.O.); 4 Graduate Program in Movement Sciences, Federal University of Mato Grosso do Sul (UFMS), Campo Grande 79070-900, MS, Brazil

**Keywords:** strength training, myostatin, muscle anabolism, creatine, muscle fibers

## Abstract

Creatine has been used to maximize resistance training effects on skeletal muscles, including muscle hypertrophy and fiber type changes. This study aimed to evaluate the impact of creatine supplementation on the myostatin pathway and myosin heavy chain (MyHC) isoforms in the slow- and fast-twitch muscles of resistance-trained rats. Twenty-eight male Wistar rats were divided into four groups: a sedentary control (Cc), sedentary creatine supplementation (Cr), resistance training (Tc), and resistance training combined with creatine supplementation (Tcr). Cc and Tc received standard commercial chow; Cr and Tcr received a 2% creatine-supplemented diet. Tc and Tcr performed a resistance training protocol on a ladder for 12 weeks. Morphology, MyHC isoforms, myostatin, follistatin, and ActRIIB protein expressions were analyzed in soleus and white gastrocnemius portion samples. The results were analyzed using two-way ANOVA and Tukey’s test. Tc and Tcr exhibited higher performance than their control counterparts. Resistance training increased the ratio between muscle and body weight, the cross-sectional area, as well as the interstitial collagen fraction. Resistance training alone increased MyHC IIx and follistatin while reducing myostatin (*p* < 0.001) and ActRIIB (*p* = 0.040) expressions in the gastrocnemius. Resistance training induced skeletal muscle hypertrophy and interstitial remodeling, which are more evident in the gastrocnemius muscle. The effects were not impacted by creatine supplementation.

## 1. Introduction

In the diverse world of commercial supplements, creatine monohydrate stands out as being widely used by high-level athletes and physically active individuals [1,2,3]. Creatine monohydrate consumption can impact skeletal muscle through multiple mechanisms which sustain muscle remodeling [4]. Creatine supplementation is commonly used to maximize the effects of resistance training, including neuromuscular recruitment, increased protein synthesis, and muscle fiber type changes [5,6,7,8,9].

Combinations between exercise training and creatine interventions resulted in greater lean mass and lower muscle protein catabolism [10,11]. Another possible effect of creatine supplementation is the adaptive modulation of myosin heavy chain (MyHC) isoforms in skeletal muscle fibers [12,13,14,15,16]. MyHC isoform profiles determine the muscle fiber phenotype; in general, predominantly MyHC I fibers have greater slow twitch-oxidative characteristics, while fibers containing more MyHC II mostly have a fast glycolytic metabolism [17,18]. Skeletal muscles have a diverse proportion and distribution of highly adaptable fiber types so that phenotypical aspects can be affected by several molecular signaling pathways, which regulate protein synthesis and myogenic activity [19,20].

Myostatin, an extracellular myokine that is primarily expressed in skeletal muscle, plays a crucial role in downregulating muscle mass and modulating fiber-type composition [21,22]. Its expression is inversely associated with the amount of fast glycolytic fibers [23,24,25,26]. Myostatin signaling is complex and comprises the activation of several downstream pathways. Mature myostatin binds to the Type IIB activin receptor (ActRIIB) and initiates signaling cascades that upregulate the genes involved in atrophy and downregulate genes involved in myogenesis. Among potential myostatin inhibitors, follistatin is an antagonist of transforming growth factor- β (TGF-β) family members, inhibiting the link of myostatin to ActRIIB. In this context, follistatin overexpression can be associated with skeletal muscle hypertrophy [21,27].

Resistance exercise training has been associated with reduced serum myostatin levels, as well as follistatin modulation [28] and increased muscle mass; these effects were amplified in response to creatine supplementation [29]. In vitro studies have shown that creatine prevents or reverses myostatin-induced muscle atrophy and increases the expression of myostatin-negative regulatory genes [30]. In pigs and chickens, creatine supplementation increased myogenic factor expression and reduced myostatin mRNA levels [31,32]. However, the impact of a combination of resistance training and creatine supplementation on morphology, MyHC isoform expression, and myostatin pathway signaling in skeletal muscles has not been clarified.

The aim of this study was to evaluate the effects of creatine monohydrate supplementation on morphology, MyHC isoform expression, and myostatin pathway signaling in gastrocnemius (white portion) and the soleus muscles of rats submitted to resistance training. Since the gastrocnemius muscle superficial area (white portion) is characterized by the predominance of fast-twitch fibers, while the soleus is a classical slow-twitch muscle, these muscles were used in this study. We hypothesized that a combination of creatine monohydrate supplementation and resistance training could attenuate myostatin expression and modulate downstream targets, promoting more accentuated changes in white gastrocnemius than in the soleus muscle.

## 2. Materials and Methods

### 2.1. Animal and Experimental Design

Male Wistar rats (60 days old) were assigned to four groups: a sedentary control (Cc, *n* = 7), sedentary creatine supplementation (Cr, *n* = 7), resistance training (Tc, *n* = 7), and resistance training combined with creatine supplementation (Tcr, *n* = 7). Cc and Tc groups received a daily diet of commercial rodent chow (Nuvilab^®^ CR1, Curitiba, PR, Brazil; 3.6 kcal/g); Cr and Tcr groups received the same standard daily diet but supplemented with creatine monohydrate at a dosage of 2% daily diet weight (3.7 kcal/g) [33,34]. Tc and Tcr groups were submitted to a ladder-climbing resistance training protocol [35]. All animals received water ad libitum and were housed (three rats per cage) under controlled temperature and humidity conditions with 12 h light/dark cycles. The experimental protocol duration was 12 weeks.

All procedures and the experimental protocol were approved by our institution’s Animal Ethics Committee (Protocol Number 873/2017) in accordance with the Brazilian College of Animal Experimentation (COBEA) and the US National Institutes of Health “Guide for the Care and Use of Laboratory Animals” (NIH, 2011).

### 2.2. Climbing Exercise Familiarization

All trained rats were familiarized with the climbing exercise protocol for three days with a 10% animal body weight load. A total of 8 to 12 climbing sets were considered a complete session during familiarization [36,37].

### 2.3. Initial Maximal Carrying Capacity

All rats were submitted to incremental tests to determine their initial maximum carrying capacity. The climbing test was performed against a 75% animal body weight load. After a 120 s interval, a 30 g load was added to the resistance apparatus for a new climb. The test was completed when the maximal load resulted in exhaustion or the impossibility of additional climbing. The test was also stopped when eight exercise sets were completed. The highest weight carried to the top of the ladder was considered the maximal load [35,36,37,38,39].

### 2.4. Resistance Training Protocol

Resistance training groups performed resistance training three times per week during the dark cycle for 12 weeks, resulting in a total of 36 sessions. Each session consisted of four to nine climbs. The first four climbs were performed with 50%, 75%, 90%, and 100% of the maximal resistance load achieved during the incremental test. Then, 30 g loads were progressively added to each subsequent climb up to the daily limit of 9 climbs [35]. Resistance loads consisted of lead weights placed in conical plastic flasks, which were attached to the proximal part of the rat tail.

### 2.5. Final Maximal Carrying Capacity

The maximal carrying capacity test was repeated after 12 weeks to determine the final maximal performance.

### 2.6. General Characteristics, Tissue Collection, and Serum Biochemical Analysis

Food consumption was measured daily, and body weight (BW) was evaluated weekly. Calorie intake was calculated as follows: daily food consumption x diet energy density. Feed efficiency (the ability to convert calorie intake into BW) was determined by dividing BW gain (g) by the total calorie intake (Kcal) [40]. Ingested creatine was calculated from 2% of the total food intake. At the end of the experiment, the rats were submitted to 6–8 h fasting, were anesthetized with thiopental (80 mg/kg), and euthanized by decapitation. Blood was collected for biochemical analyses, and the serum was separated by centrifugation at 3000× *g* for 10 min and then stored at −80 °C for subsequent assessment. Glucose, cholesterol, triglycerides, albumin, and total protein serum levels were determined by spectrophotometry using enzymatic kits [40,41]. The gastrocnemius and soleus muscles from both pelvic limbs were quickly removed and weighed. After that, muscle samples were immediately frozen in liquid nitrogen and stored at −80 °C until analysis. The right tibia was dissected, measured using a pachymeter, and used to normalize muscle mass [41,42].

### 2.7. Skeletal Muscle Morphology

Total gastrocnemius and soleus muscle weights in both absolute values and respective ratios with body weight and tibia length were used to characterize the macroscopic morphology. Soleus and white (superficial) portions of gastrocnemius muscles were used to obtain cross-sectional histological sections (8-μm-thick) using a cryostat at −20 °C. The slides were stained with hematoxylin-eosin (HE) to assess the cross-sectional area; at least 150 fibers were measured per animal. Other histological slides were submitted to Picrosirius red (Sirius red F3BA) staining; at least 10 fields per animal were digitalized and used to calculate the interstitial collagen fraction [41,43]. Histological analyzes were performed at 400× magnification using a Leica DM5500B microscope (Wetzlar, Germany) coupled to a digital image projection video camera equipped with Leica Application Suite version 4.0.0 (Heerbrugg, Switzerland). Cross-sectional fiber areas were measured using Image J software (Wayne Rasbandat NIH, Bethesda, MD, USA), and an interstitial collagen fraction was quantified using Image Pro-plus Version 6.0.0.260 (Media Cybernetics, Rockville, MD, USA).

### 2.8. Western Blotting

Protein levels were analyzed by Western blot according to previous studies [41,42,43,44]. Protein was extracted using a RIPA buffer (1 mL/100 mg tissue) containing protease and phosphatase inhibitors. The supernatant protein content was quantified by the Bradford assay [45]. Samples were separated into a polyacrylamide gel and then transferred to a nitrocellulose membrane. After blockade with 5% skimmed milk in TBST for 1 h, the membrane was incubated overnight at 4 °C with specific antibodies: anti-myostatin (GDF-8, sc-6885-R)—dilution 1:200, anti-follistatin (sc-30194)—dilution 1:400, and anti-ActRIIB (sc-376593)—dilution 1:200, Santa Cruz Biotechnology, Inc., Santa Cruz, Dallas, TX, USA). The membrane was then washed with TBS and Tween 20 and incubated with a secondary peroxidase-conjugated antibody (90 min at room temperature). Enhanced Chemio Luminescence (Luminata Crescendo^®^ reagent, Merck Millipore; Darmstadt, Germany) was used to detect bound antibodies [41]. The membrane was then incubated with a ReBlot Plus Strong Antibody Stripping Solution, 10×—Millipore (Burlington, MA, USA), to remove the antibodies attached to the membrane. The blockade process was repeated, and the membrane was incubated overnight at 4 °C with glyceraldehyde-3-phosphate dehydrogenase (GAPDH, sc-32233, Santa Cruz Biotechnology, Inc., Dallas, TX, USA). The procedure continued as described above until a signal was detected. Afterward, respective bands were quantified by densitometry using a Gel-Pro Analyzer 3.1. The results obtained for each protein were normalized to those obtained for GAPDH.

### 2.9. Myosin Heavy Chain Isoforms

MyHC isoforms were quantified after electrophoresis in a sodium dodecyl sulfate-polyacrylamide gel (SDS-PAGE) [46,47]. Frozen samples were mechanically homogenized in a protein extraction solution containing a 50 mM phosphate potassium lysis buffer 0.5 mL/50 mg tissue. The total protein quantification was performed in supernatant aliquots by the Bradford method. Small volumes of the diluted extracts (10 μL) were loaded onto a 7–10% SDS-PAGE separating gel with a 4% stacking gel, which was run overnight (27 h) at 70 V and stained with Coomassie blue R (Sigma-Aldrich^®^, St. Louis, MO, USA). MyHC isoforms were quantified by densitometry and identified based on predominant fiber types in studied muscles samples, as reported previously [48,49,50]. MyHC I and MyHC IIa isoforms were observed in the soleus muscle, and MyHC IIx and MyHC IIb isoforms were found in the gastrocnemius white portion. The relative quantity was expressed as a percentage of the total MyHC expression.

### 2.10. Statistical Analysis

The results were expressed as the mean ± standard deviations. The data distribution was analyzed using the Kolmogorov–Smirnov test. Student’s t-test was used to compare creatine intake between Cr and Tcr groups. Other variables were evaluated using a two-way analysis of variance (Two-Way ANOVA). When significant differences were found (*p* < 0.05) post hoc, Tukey’s multiple comparisons test was performed. The level of significance was 5%.

## 3. Results

### 3.1. Maximal Carrying Capacity

**Figure 1:**
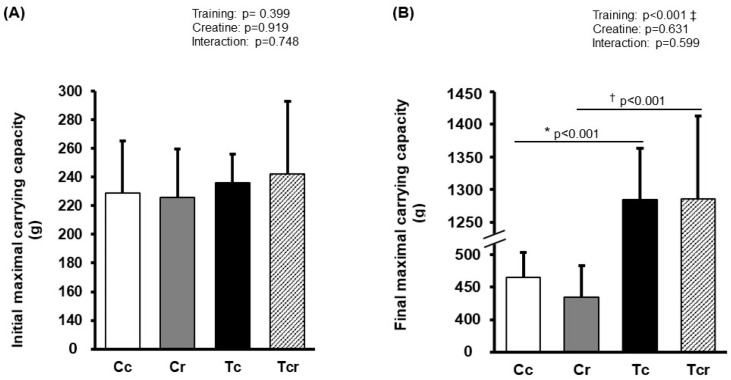
Maximum carrying capacity in climbing test before (**A**) and after (**B**) the training protocol. Cc (*n* = 7): sedentary control; Cr (*n* = 7): sedentary creatine supplementation; Tc (*n* = 7): resistance training; Tcr (*n* = 7): resistance training combined with creatine supplementation. Two-way ANOVA and Tukey test. ‡ *p* < 0.05, resistance training effect; * *p* < 0.05 vs. Cc; † *p* < 0.05 vs. Cr.

Before the training protocol, experimental groups exhibited similar performance during the maximal carrying capacity test (Cc 228 ± 36; Cr 225 ± 33; Tc 236 ± 19; Tcr 242 ± 25 g; *p* > 0.05). After training, Tc and Tcr presented a higher load-bearing capacity than their respective controls (Cc 465 ± 38; Cr 434 ± 48; Tc 1284 ± 79; Tcr 1285 ± 127 g; Figure 1).

### 3.2. Nutritional and Serum Biochemical Data

The initial body weight was not different between the groups. At the end of the experimental protocol, exercise training intervention resulted in a lower final body weight; Tc exhibited reduced body weight when compared to the Cc and Tcr groups. The total caloric intake did not differ between groups. Feed efficiency was lower in Tc and Tcr than Cc and Cr, respectively, and was higher in Cr than Cc. Creatine intake did not differ between Cr and Tcr groups (Table 1).

Relative to the serum biochemical data, although groups exhibited similar responses in terms of glycemia, cholesterol, albumin, and protein values, exercise training intervention was independently associated with lower triglyceride serum levels (Sedentary groups, 97.4 ± 1.7; Trained groups, 92.6 ± 1.7 mg/dL; *p* < 0.05, Table 1).

### 3.3. Morphological Characterization

The total gastrocnemius mass, in absolute values or normalized by the tibia length, did not differ between the groups. The gastrocnemius weight-to-body weight ratio was increased by resistance exercise training; Tc showed a higher gastrocnemius weight-to-body weight ratio than Cc. Soleus mass, in absolute values and in ratios with body weight and tibia length, did not differ between the groups. (Table 2).

**Figure 2:**
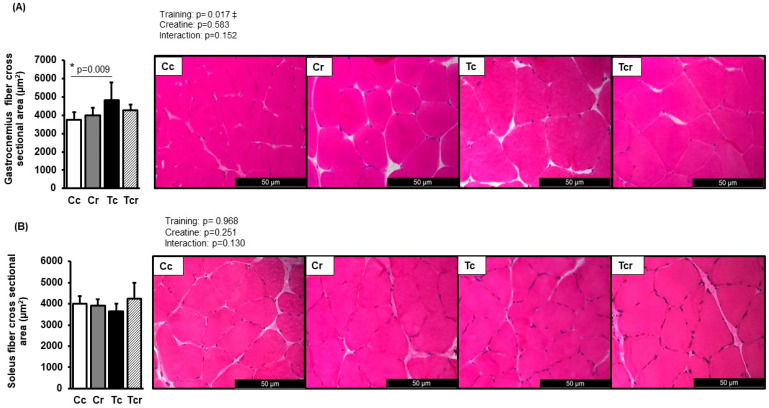
Cross-sectional area and representative transverse histological sections of the gastrocnemius (**A**) and soleus (**B**) muscles (400-fold magnification) stained with hematoxylin-eosin. Cc (*n* = 7): sedentary control; Cr (*n* = 7): sedentary creatine supplementation; Tc (*n* = 7): resistance training; Tcr (*n* = 7): resistance training combined with creatine supplementation. Two-way ANOVA and Tukey test. ‡ *p* < 0.05, resistance training effect; * *p* < 0.05 vs. Cc.

Representative pictures of hematoxylin-eosin-stained muscle transverse sections are shown in Figure 2. The cross-sectional area of fibers from the gastrocnemius white portion was increased in response to resistance exercise training (Cc 3731 ± 415; Cr 3977 ± 422; Tr 4813 ± 982; Tcr 4273 ± 305 µm^2^, Figure 2A). Tc exhibited a higher cross-sectional area than the Cc group. On the other hand, soleus cross-sectional areas did not differ between the groups (Cc 3997 ± 353; Cr 3910 ± 289; Tr 3642 ± 360; Tcr 4247 ± 736 µm^2^, Figure 2B).

**Figure 3:**
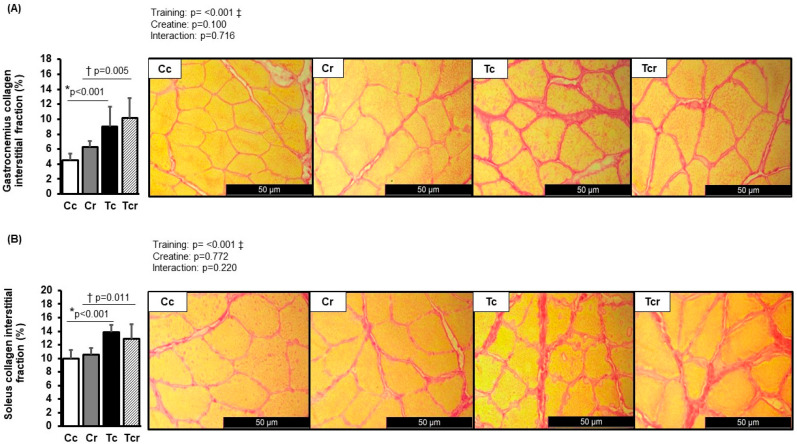
Collagen interstitial fraction and representative transverse histological sections from gastrocnemius (**A**) and soleus (**B**) muscles (400-fold magnification) stained with Picrosirius red. Cc (*n* = 7): sedentary control; Cr (*n* = 7): sedentary creatine supplementation; Tc (*n* = 7): resistance training; Tcr (*n* = 7): resistance training combined with creatine supplementation. Two-way ANOVA and Tukey test. ‡ *p* < 0.05, resistance training effect; * *p* < 0.05 vs. Cc; † *p* < 0.05 vs. Cr.

Picrosirius red-stained muscle transverse sections are shown in Figure 3. Collagen interstitial fraction was increased in response to resistance training in the soleus (Cc 9.97 ± 1.25; Cr 10.55 ± 0.98; Tr 13.85 ± 1.04; Tcr 12.92 ± 2.07%) and gastrocnemius white portion (Cc 4.50 ± 0.91; Cr 6.25 ± 0.86; Tr 9.05 ± 2.62; Tcr 10.18 ± 2.60%) muscles. Tr and Tcr presented a higher collagen interstitial fraction than their respective controls.

### 3.4. Myosin Heavy Chain Isoforms Distribution

**Figure 4:**
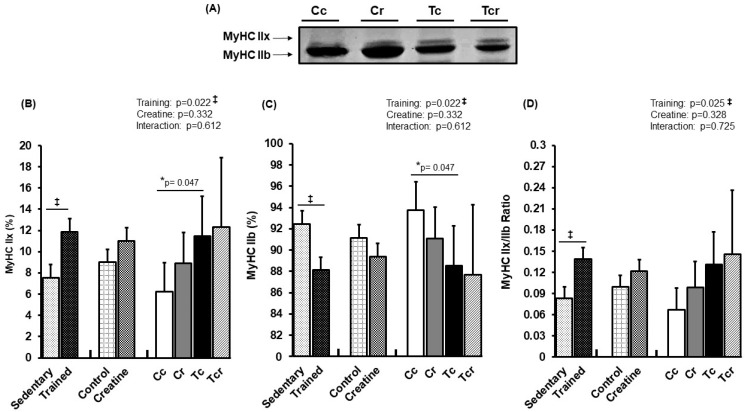
Representative bands of myosin heavy chain isoforms (MyHC IIx and MyHC IIb) (**A**); relative proportions (%) of MyHC IIx (**B**), and MyHC IIb (**C**) isoforms, and MyHC IIx/MyHC IIb ratio (**D**) in the gastrocnemius muscle. Isolated factors: Sedentary; Trained; Control; Creatine. Groups: Cc (*n* = 6): sedentary control; Cr (*n* = 6): sedentary creatine supplementation; Tc (*n* = 6): resistance training; Tcr (*n* = 6): resistance training combined with creatine supplementation. Two-way ANOVA and Tukey test. ‡ *p* < 0.05, resistance training effect; * *p* < 0.05 vs. Cc.

**Figure 5:**
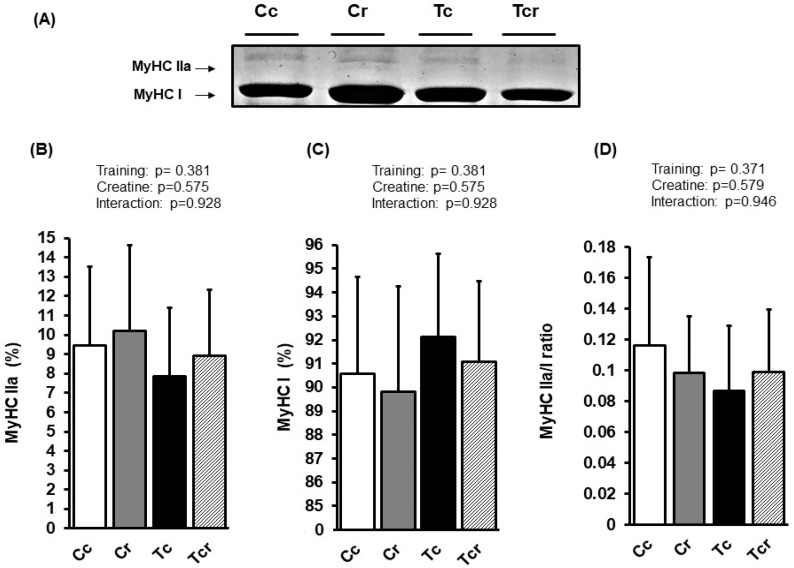
Representative bands of myosin heavy chain isoforms (MyHC IIa and MyHC I) (**A**); relative proportions (%) of MyHC IIa (**B**), and MyHC I (**C**) isoforms, and MyHC IIa/MyHC I ratio (**D**) in the soleus muscle. Groups: Cc (*n* = 6): sedentary control; Cr (*n* = 6): sedentary creatine supplementation; Tc (*n* = 6): resistance training; Tcr (*n* = 6): resistance training combined with creatine supplementation. Two-way ANOVA (*p* > 0.05).

Figure 4 and Figure 5 show MyHC isoform expressions in the gastrocnemius white portion and soleus muscles, respectively. Gastrocnemius showed significant effects from exercise training alone (*p* = 0.022); MyHC IIx expression increased in response to resistance training (Cc 6.25 ± 2.68; Cr 8.88 ± 2.94; Tc 11.47 ± 3.73; Tcr 12.30 ± 6.58%). MyHC IIb expression was lower in the trained groups than in the controls (Cc 93.75 ± 2.68; Cr 91.12 ± 2.94; Tc 88,53 ± 3.73; Tcr 87.70 ± 6.58%).

The soleus muscle’s MyHC IIa isoform expression was similar between the groups (Cc 9.43 ± 4.08; Cr 10.19 ± 4.43, Tc 7.87 ± 3.50, Tcr 8.92 ± 3.40%). Likewise, the MyHC I isoform was not affected by dietary intervention or resistance exercise training (Cc 90.57 ± 4.08; Cr 89.81 ± 4.43, Tc 92.13 ± 3.50, Tcr 91.08 ± 3.40%; Figure 5).

### 3.5. Protein Expression

**Figure 6:**
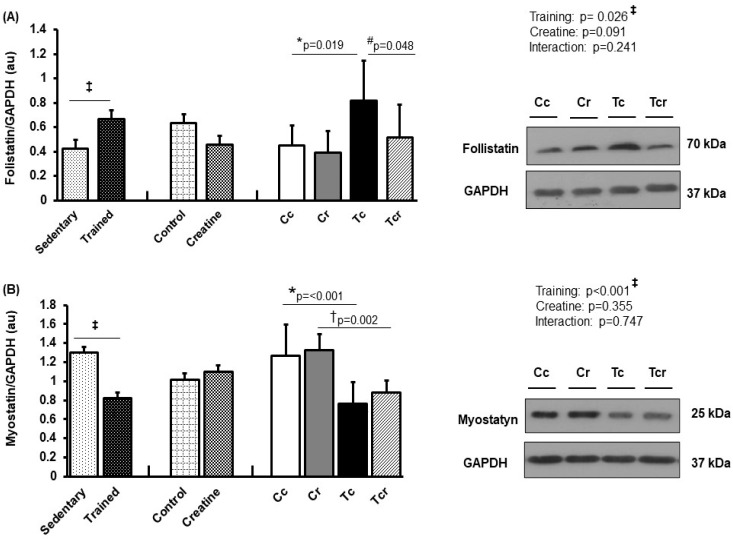
Protein levels and representative Western blots of follistatin (**A**) and myostatin (**B**) expression in the gastrocnemius muscle. Protein levels were normalized to glyceraldehyde-3-phosphate dehydrogenase (GAPDH). Isolated factors: Sedentary; Trained; Control; Creatine. Groups: Cc (*n* = 7): sedentary control; Cr (*n* = 7): sedentary creatine supplementation; Tc (*n* = 7): resistance training; Tcr (*n* = 7): resistance training combined with creatine supplementation. Two-way ANOVA and Tukey test. ‡ *p* < 0.05, resistance training effect; * *p* < 0.05 vs. Cc; † *p* < 0.05 vs. Cr; # *p* < 0.05 vs. Tc.

**Figure 7:**
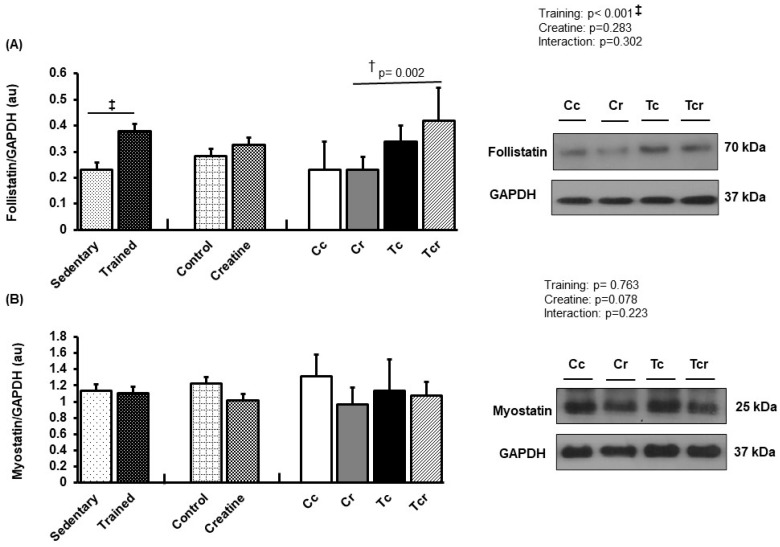
Protein levels and representative Western blots of follistatin (**A**) and myostatin (**B**) expression in the soleus muscle. Protein levels were normalized to glyceraldehyde-3-phosphate dehydrogenase (GAPDH). Isolated factors: Sedentary; Trained; Control; Creatine. Groups: Cc (*n* = 7): sedentary control; Cr (*n* = 7): sedentary creatine supplementation; Tc (*n* = 7): resistance training; Tcr (*n* = 7): resistance training combined with creatine supplementation. Two-way ANOVA and Tukey test. ‡ *p* < 0.05, resistance training effect; † *p* < 0.05 vs. Cr.

Resistance exercise training increased follistatin expression in the gastrocnemius white portion (Figure 6A) and soleus muscles (Figure 7A). Gastrocnemius follistatin expression was higher in Tc than in the Cc and Tcr groups (Cc 0.45 ± 0.16; Cr 0.40 ± 0.17, Tc 0.82 ± 0.33, Tcr 0.52 ± 0.27 arbitrary units; Figure 6A). Both trained groups presented lower gastrocnemius myostatin levels than their respective controls (Cc 1.27 ± 0.32; Cr 1.32 ± 0.17, Tc 0.76 ± 0.22, Tcr 0.88 ± 0.13 arbitrary units; Figure 6B).

Soleus follistatin was higher in Tcr than Cr (Cc 0.23 ± 0.11; Cr 0.23 ± 0.04, Tc 0.34 ± 0.06, Tcr 0.42 ± 0.12 arbitrary units; Figure 7A), and myostatin expression did not differ between the groups (Cc 1.31 ± 0.27; Cr 0.96 ± 0.21, Tc 1.13 ± 0.38, Tcr 1.07 ± 0.17 arbitrary units; Figure 7B).

**Figure 8:**
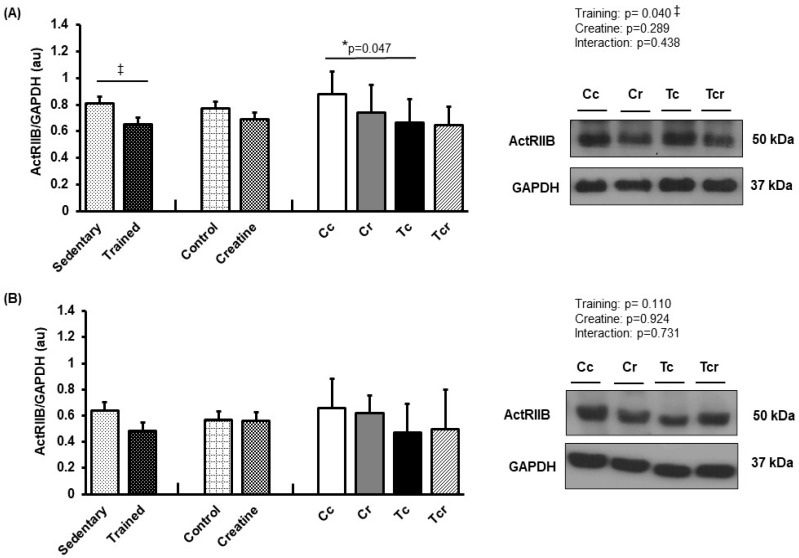
Protein levels and representative Western blots of ActRIIB in (**A**) gastrocnemius and (**B**) the soleus muscle. Protein levels were normalized to glyceraldehyde-3-phosphate dehydrogenase (GAPDH). Isolated factors: Sedentary; Trained; Control; Creatine. Groups: Cc (*n* = 7): sedentary control; Cr (*n* = 7): sedentary creatine supplementation; Tc (*n* = 7): resistance training; Tcr (*n* = 7): resistance training combined with creatine supplementation. Two-way ANOVA and Tukey test. ‡ *p* < 0.05, resistance training effect; * *p* < 0.05 vs. Cc.

Resistance training alone reduced ActRIIB expression in the gastrocnemius white portion muscle (Cc 0.88 ± 0.17; Cr 0.74 ± 0.20, Tc 0.66 ± 0.17, Tcr 0.64 ± 0.14 arbitrary units; Figure 8A). Soleus ActRIIB expression did not differ between the groups (Cc 0.66 ± 0.22; Cr 0.62 ± 0.13, Tc 0.47 ± 0.21, Tcr 0.50 ± 0.29 arbitrary units; Figure 8B).

## 4. Discussion

The current study aimed to evaluate the effects of creatine monohydrate supplementation when combined with resistance training on the soleus, a classic slow-twitch muscle, and gastrocnemius white portion, an important fast-twitch muscle type. In contrast to our hypothesis, creatine supplementation, both alone or combined with resistance training, had little impact on morphological and molecular features in both skeletal muscle types. On the other hand, the resistance exercise training intervention resulted in morphological and molecular adaptive effects in both skeletal muscles; these effects were more accentuated in the gastrocnemius than in the soleus muscle, partially confirming the primary hypothesis.

**Figure 9:**
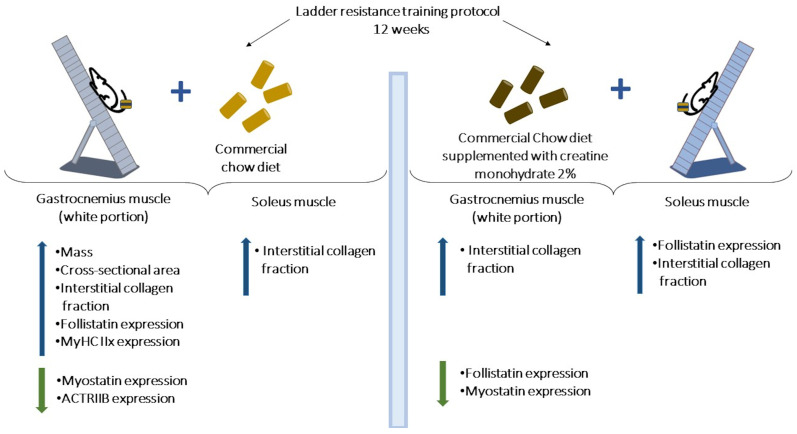
Differential effects of ladder resistance training and creatine monohydrate supplementation on gastrocnemius (white portion) and soleus muscles. Resistance training-induced skeletal muscle remodeling is greater in the fast-twitch than the slow-twitch muscle type.

Indeed, resistance exercise training is a non-pharmacological intervention that, when performed continuously and safely, can result in metabolic benefits and promote physiological and structural adaptations that are associated with improved neuromuscular function and motor performance [51,52,53,54,55]. In this study, resistance training was associated with lower triglyceride serum levels and an increased maximal load-carrying capacity, confirming the primary premise as well as findings derived from animal models [56,57]. Moreover, the 12 weeks resistance training protocol increased the mass, cross-sectional area, collagen interstitial fraction, MyHC IIx proportion, and follistatin expression while reducing myostatin and ActRIIB protein levels in the gastrocnemius white portion muscle. Likewise, exercise training resulted in a greater collagen interstitial fraction in the soleus muscle. When combined with creatine supplementation, the ladder resistance training protocol reduced the follistatin level expression in the gastrocnemius muscle (Figure 9).

Skeletal muscle is highly adaptable to increased muscle load, presenting compensatory hypertrophy with improved strength [51,58,59]. In accordance with our results, Ribeiro et al. [60] observed that resistance training on a ladder (3×/week for 12 weeks) induced hypertrophy that was restricted to the gastrocnemius muscle in three-month-old rats; the cross-sectional area in the soleus muscle was unaffected by the intervention. Based on a similar protocol, Deschenes et al. [61] failed to show a significant increase in the cross-sectional area of soleus muscle fibers after resistance training for six weeks. By contrast, rodents that submitted to an alternative training protocol (16 weeks; 5×/week) exhibited a greater soleus cross-sectional area [62]. This is perhaps because soleus remodeling can be associated with greater volume and duration protocols. It is noteworthy that resistance training is predominantly associated with fast-twitch Type II fibers hypertrophy in the skeletal muscle [51,63,64,65]. Type II muscle fibers respond preferentially to intense training protocols, while Type I muscles are more susceptible to high-volume exercise [66]. Besides these morphological effects, the muscle recruitment pattern in response to exercise training may also be associated with different MyHC stimuli. Accordingly, resistance training preferentially impacted the MyHC IIx proportion in gastrocnemius white portion muscles with no changes in the soleus muscle composition (Figure 4 and Figure 5).

Additionally, resistance training promoted extensive interstitial remodeling in both skeletal muscle types. Recently, Braggion et al. [67] also observed that training performed on a ladder (3×/week for 12 weeks) increased collagen fibers in the soleus skeletal muscle of ovariectomized rats. The upregulation of collagen turnover due to prolonged training could be caused by the increased activity of fibroblasts involved in muscle hypertrophy and regeneration [67,68]. This change may provide mechanical support to muscle fibers, as resistance training promotes a high contractile demand to multiple skeletal muscles, despite different histological, biochemical, and metabolic properties [51].

Considering these molecular mechanisms, resistance training reduced myostatin expression in the gastrocnemius white portion. Myostatin, also known as growth/differentiation factor-8 (GDF-8), is a TGF-β family member [21]. While myostatin overexpression reduces muscle mass, myostatin null animals have an increased muscle mass [69,70]. This effect has been observed with natural myostatin mutations in animals and humans [71,72,73,74]. Supporting the findings of our study, myostatin mRNA expression decreased in elderly women after resistance training [75]. Hayashi et al. [76] described how increased myostatin was associated with the reduced expression of the fast MyHC IIx isoform in cattle. Likewise, Ribeiro et al. [60] observed reduced myostatin levels in male rat gastrocnemius in response to 12-week ladder resistance training. Relative to follistatin expression, the results of this study confirm previous evidence [77,78]; resistance exercise training positively modulates follistatin and negatively modulates myostatin and ActRIIB, which are associated with gastrocnemius hypertrophy. In addition to inhibiting myostatin binding to its receptor, follistatin may have anabolic activity, which depends on insulin-like growth factor-1 or insulin [79]. In this context, creatine supplementation could improve the activity of components in the insulin signaling pathway [80]. This possibility may have supported the higher follistatin expression in the soleus skeletal muscle (Figure 7) of trained rats that were creatine supplemented.

In general, exercise resistance training effects are frequently accentuated by creatine supplementation [4]. In previous studies, creatine supplementation promoted an increase in muscle mass during resistance training with a progressive overload increase [12,81]. However, Cooke et al. [82] observed that 12 weeks of creatine supplementation did not modify the lean mass, muscle strength, total myofibrillar protein content, and/or muscle fiber cross-sectional area in men undergoing resistance training. Similarly, creatine supplementation did not promote any direct anabolic effect on the skeletal muscles of rats that were submitted to 5 weeks of intense jumping resistance training [48]. The authors argued for the possible benefits of creatine supplementation in terms of hypertrophic mechanisms activation and additional muscle mass gain in trained rats, which was dependent on a higher workload [48].

Based on the principle of specificity, physiological adaptations due to resistance exercise training are dependent on specific stress characteristics [83]. From this perspective, potential effects depend on a combination of training variables and, generally, are associated with metabolic stress due to glycolysis overload [52,84]. Although creatine monohydrate supplementation could contribute to improvements in glycolysis, it is predominantly beneficial to increase muscle mass through the creatine and phosphocreatine systems [83]. Thus, any anabolic effect from creatine ingestion could be more pronounced when associated with ladder resistance training protocols based on greater workloads resulting from increases in rest periods as well as the number of sets within a session and/or frequency of workouts per week. Therefore, a mismatch between exercise training demand and nutritional supplementation may not be discarded as a possible background for the little effect of creatine consumption in this study. Further investigation is needed to clarify the potential creatine-induced molecular mechanisms modulating the skeletal muscle phenotype.

Moreover, although lactate concentration is commonly used to determine resistance training intensity, this variable was not evaluated to determine functional capacity, which can be considered an additional limitation in this study.

## 5. Conclusions

Resistance exercise training induces skeletal muscle hypertrophy and interstitial remodeling in rats, which is more evident in the gastrocnemius muscle white portion than in the soleus muscle. This adaptive response can be associated with the myostatin signaling pathway modulation and increased MyHC IIx expression and is not affected by creatine supplementation.

## Figures and Tables

**Table 1:** Nutritional and serum biochemical data.

Variables		Groups				Factors (*p*-Value)		
		Cc	Cr	Tc	Tcr	Training	Creatine	Interaction
Nutritional	Initial body weight (g)	240 ± 22	242 ± 25	237 ± 20	251 ± 29	0.740	0.409	0.547
	Final body weight (g)	392 ± 34	407 ± 34	350 ± 71 *	390 ± 72 #	0.044 ‡	0.052	0.374
	Food intake (g)	2132 ± 150	2016 ± 82	2022 ± 160	1983 ± 169	0.162	0.130	0.444
	Creatine intake (g)	**-**	40.33 ± 1.64	**-**	39.67 ± 2.32	0.551	-	-
	Calorie intake (Kcal)	7699 ± 544	7421 ± 302	7301 ± 578	7325 ± 444	0.186	0.489	0.414
	Feed efficiency (mg/Kcal)	1.9 ± 0.1	2.2 ± 0.1 *	1.7 ± 0.2 *	1.9 ± 0.1 †	<0.001 ‡	0.007 §	0.437
Biochemical	Glucose (mg/dL)	134 ± 17	131 ± 24	133 ± 24	144 ± 21	0.492	0.629	0.410
	Total cholesterol (mg/dL)	71.3 ± 9.1	77.8 ± 16.6	78.6 ± 9.3	76.5 ± 6.4	0.481	0.600	0.309
	HDL cholesterol (mg/dL)	38.7 ± 6.2	45.1 ± 8.2	42.1 ± 4.3	42.4 ± 4.2	0.880	0.156	0.188
	Non-HDL (mg/dL)	33.9 ± 5.4	32.8 ± 11.4	36.2 ± 8.5	34.1 ± 2.8	0.537	0.602	0.858
	Triglycerides (mg/dL)	95.8 ± 7.7	98.9 ± 6.3	91.0 ± 3.4	94.2 ± 6.4	0.049 ‡	0.190	0.983
	Albumine (mg/dL)	2.30 ± 0.15	2.30 ± 0.12	2.27 ± 0.17	2.44 ± 0.11	0.291	0.119	0.119
	Total protein (mg/dL)	5.16 ± 2.24	5.93 ± 0.41	6.13 ± 0.54	6.46 ± 0.35	0.107	0.231	0.625

HDL: high-density lipoprotein cholesterol; Cc: sedentary control; Cr: creatine supplementation; Tc: resistance training; Tcr: resistance training and creatine supplementation. Mean ± SD. Two-way ANOVA and Tukey test. ‡ *p* < 0.05, resistance training effect; § *p* < 0.05, creatine effect; * *p* < 0.05 vs. Cc; # *p* < 0.05 vs. Tc; † *p* < 0.05 vs. Cr. Creatine intake results analyzed by Student’s *t*-test (*p* > 0.05).

**Table 2:** Macroscopic muscle morphology.

Variables	Groups				Factors (*p*-Value)		
	Cc	Cr	Tc	Tcr	Training	Creatine	Interaction
Gastrocnemius (mg)	2255 ± 172	2378 ± 106	2433 ± 572	2531 ± 463	0.262	0.451	0.931
Gastrocnemius/BW (mg/g)	5.76 ± 0.24	5.86 ± 0.34	6.92 ± 1.35 *	6.52 ± 1.30	0.020 ‡	0.676	0.498
Gastrocnemius/tibia (mg/mm)	52.9 ± 3.8	56.6 ± 1.6	59.0 ± 13.0	59.7 ± 11.0	0.181	0.512	0.646
Soleus (mg)	141 ± 19	146 ± 20	125 ± 26	153 ± 30	0.633	0.095	0.241
Soleus/BW (mg/g)	0.36 ± 0.03	0.36 ± 0.03	0.35 ± 0.05	0.39 ± 0.05	0.463	0.359	0.307
Soleus/tibia (mg/mm)	3.33 ± 0.47	3.50 ± 0.46	3.05 ± 0.60	3.60 ± 0.62	0.677	0.097	0.362

Cc: sedentary control; Cr: creatine supplementation; Tc: resistance training; Tcr: resistance training and creatine supplementation. Mean ± SD. Two-way ANOVA and Tukey test. ‡ *p* < 0.05, resistance training effect; * *p* < 0.05 vs. Cc.

## Data Availability

All data that support the findings of this study are available from the corresponding author on reasonable request.

## References

[1] Butts J., Jacobs B., Silvis M. (2017). Creatine Use in Sports. Sport. Health Multidiscip. Approach.

[2] Terjung R.L., Clarkson P., Eichner E.R., Greenhaff P.L., Hespel P.J., Israel R.G., Kraemer W.J., Meyer R.A., Spriet L.L., Tarnopolsky M.A. (2000). Physiological and Health Effects of Oral Creatine Supplementation. Med. Sci. Sports Exerc..

[3] Wax B., Kerksick C.M., Jagim A.R., Mayo J.J., Lyons B.C., Kreider R.B. (2021). Creatine for Exercise and Sports Performance, with Recovery Considerations for Healthy Populations. Nutrients.

[4] Pinder M.A., Myrie S.B. (2017). Creatine Supplementation and Skeletal Muscle Metabolism for Building Muscle Mass-Review of the Potential Mechanisms of Action. Curr. Protein Pept. Sci..

[5] Verdijk L.B., Gleeson B.G., Jonkers R.A.M., Meijer K., Savelberg H.H.C.M., Dendale P., van Loon L.J. (2009). Skeletal Muscle Hypertrophy Following Resistance Training Is Accompanied by a Fiber Type-Specific Increase in Satellite Cell Content in Elderly Men. J. Gerontol. Ser. A Biol. Sci. Med. Sci..

[6] Mitchell C.J., Churchward-Venne T.A., Bellamy L., Parise G., Baker S.K., Phillips S.M. (2013). Muscular and Systemic Correlates of Resistance Training-Induced Muscle Hypertrophy. PLoS ONE.

[7] Chilibeck P.D., Kaviani M., Candow D.G., Zello G.A. (2017). Effect of creatine supplementation during resistance training on lean tissue mass and muscular strength in older adults: A meta-analysis. Open Access J. Sport. Med..

[8] Bonilla D.A., Kreider R.B., Petro J.L., Romance R., García-Sillero M., Benítez-Porres J., Vargas-Molina S. (2021). Creatine Enhances the Effects of Cluster-Set Resistance Training on Lower-Limb Body Composition and Strength in Resistance-Trained Men: A Pilot Study. Nutrients.

[9] Nouri H., Sheikholeslami-Vatani D., Moloudi M.R. (2021). Changes in UPR-PERK pathway and muscle hypertrophy following resistance training and creatine supplementation in rats. J. Physiol. Biochem..

[10] Pinto C.L., Botelho P.B., Carneiro J.A., Mota J.F. (2016). Impact of creatine supplementation in combination with resistance training on lean mass in the elderly. J. Cachex-Sarcopenia Muscle.

[11] Aguiar A.F., Januário R.S.B., Junior R.P., Gerage A., Pina F.L.C., Nascimento M.A.D., Padovani C.R., Cyrino E. (2012). Long-term creatine supplementation improves muscular performance during resistance training in older women. Eur. J. Appl. Physiol..

[12] Willoughby D.S., Rosene J. (2001). Effects of oral creatine and resistance training on myosin heavy chain expression. Med. Sci. Sport. Exerc..

[13] Taes Y.E., Speeckaert M., Bauwens E., De Buyzere M.R., Libbrecht J., Lameire N.H., Delanghe J.R. (2004). Effect of Dietary Creatine on Skeletal Muscle Myosin Heavy Chain Isoform Expression in an Animal Model of Uremia. Nephron Exp. Nephrol..

[14] Gallo M., Gordon T., Syrotuik D., Shu Y., Tyreman N., MacLean I., Kenwell Z., Putman C.T. (2006). Effects of long-term creatine feeding and running on isometric functional measures and myosin heavy chain content of rat skeletal muscles. Pflug. Arch. Eur. J. Physiol..

[15] Gallo M., MacLean I., Tyreman N., Martins K.J.B., Syrotuik D., Gordon T., Putman C.T. (2008). Adaptive responses to creatine loading and exercise in fast-twitch rat skeletal muscle. Am. J. Physiol. Integr. Comp. Physiol..

[16] Aguiar A.F., Aguiar D.H., Felisberto A.D., Carani F.R., Milanezi R.C., Padovani C.R., Dal-Pai-Silva M. (2010). Effects of Creatine Supplementation During Resistance Training on Myosin Heavy Chain (MHC) Expression in Rat Skeletal Muscle Fibers. J. Strength Cond. Res..

[17] Staron R.S., Kraemer W.J., Hikida R.S., Fry A.C., Murray J.D., Campos G.E.R. (1999). Fiber type composition of four hindlimb muscles of adult Fisher 344 rats. Histochem..

[18] Bloemberg D., Quadrilatero J. (2012). Rapid Determination of Myosin Heavy Chain Expression in Rat, Mouse, and Human Skeletal Muscle Using Multicolor Immunofluorescence Analysis. PLoS ONE.

[19] Wigmore P.M., Evans D.J. (2002). Molecular and cellular mechanisms involved in the generation of fiber diversity during myogenesis. Int. Rev. Cytol..

[20] Schiaffino S., Reggiani C. (2011). Fiber Types in Mammalian Skeletal Muscles. Physiol. Rev..

[21] Elkina Y., Von Haehling S., Anker S.D., Springer J. (2011). The role of myostatin in muscle wasting: An overview. J. Cachexia Sarcopenia Muscle.

[22] Chen M.-M., Zhao Y.-P., Zhao Y., Deng S.-L., Yu K. (2021). Regulation of Myostatin on the Growth and Development of Skeletal Muscle. Front. Cell Dev. Biol..

[23] Artaza J.N., Bhasin S., Mallidis C., Taylor W., Ma K., Gonzalez-Cadavid N.F. (2002). Endogenous expression and localization of myostatin and its relation to myosin heavy chain distribution in C2C12 skeletal muscle cells. J. Cell. Physiol..

[24] Hennebry A., Berry C., Siriett V., O’Callaghan P., Chau L., Watson T., Sharma M., Kambadur R. (2009). Myostatin regulates fiber-type composition of skeletal muscle by regulating MEF2 and MyoD gene expression. Am. J. Physiol.-Cell Physiol..

[25] Wang M., Yu H., Kim Y.S., Bidwell C.A., Kuang S. (2012). Myostatin facilitates slow and inhibits fast myosin heavy chain expression during myogenic differentiation. Biochem. Biophys. Res. Commun..

[26] Xing X.-X., Xuan M.-F., Jin L., Guo Q., Luo Z.-B., Wang J.-X., Luo Q.-R., Zhang G.-L., Cui C.-D., Cui Z.-Y. (2017). Fiber-type distribution and expression of myosin heavy chain isoforms in newborn heterozygous myostatin-knockout pigs. Biotechnol. Lett..

[27] Han X., Møller L.L.V., De Groote E., Bojsen-Møller K.N., Davey J., Henríquez-Olguin C., Li Z., Knudsen J.R., Jensen T.E., Madsbad S. (2019). Mechanisms involved in follistatin-induced hypertrophy and increased insulin action in skeletal muscle. J. Cachex-Sarcopenia Muscle.

[28] Domin R., Dadej D., Pytka M., Zybek-Kocik A., Ruchała M., Guzik P. (2021). Effect of Various Exercise Regimens on Selected Exercise-Induced Cytokines in Healthy People. Int. J. Environ. Res. Public Health.

[29] Saremi A., Gharakhanloo R., Sharghi S., Gharaati M., Larijani B., Omidfar K. (2010). Effects of oral creatine and resistance training on serum myostatin and GASP-1. Mol. Cell. Endocrinol..

[30] Mobley C.B., Fox C.D., Ferguson B.S., Amin R.H., Dalbo V.J., Baier S., Rathmacher J.A., Wilson J.M., Roberts M.D. (2014). L-leucine, beta-hydroxy-beta-methylbutyric acid (HMB) and creatine monohydrate prevent myostatin-induced Akirin-1/Mighty mRNA down-regulation and myotube atrophy. J. Int. Soc. Sport. Nutr..

[31] Young J., Bertram H., Theil P., Petersen A.-G., Poulsen K., Rasmussen M., Malmendal A., Nielsen N., Vestergaard M., Oksbjerg N. (2007). In vitro and in vivo studies of creatine monohydrate supplementation to Duroc and Landrace pigs. Meat Sci..

[32] Chen J., Wang M., Kong Y., Ma H., Zou S. (2011). Comparison of the novel compounds creatine and pyruvateon lipid and protein metabolism in broiler chickens. Animal.

[33] Rooney K., Bryson J., Phuyal J., Denyer G., Caterson I., Thompson C. (2002). Creatine supplementation alters insulin secretion and glucose homeostasis in vivo. Metab. Clin. Exp..

[34] Ju J.-S., Smith J.L., Oppelt P.J., Fisher J.S. (2005). Creatine feeding increases GLUT4 expression in rat skeletal muscle. Am. J. Physiol. Endocrinol. Metab..

[35] Hornberger T.A., Farrar R.P. (2004). Physiological Hypertrophy of the FHL Muscle Following 8 Weeks of Progressive Resistance Exercise in the Rat. Can. J. Appl. Physiol..

[36] Medeiros C.S., Neto I.V.d.S., Silva K.K.S., Cantuária A.P.C., Rezende T.M.B., Franco O.L., Marqueti R.d.C., Freitas-Lima L.C., Araujo R.C., Yildirim A. (2021). The Effects of High-Protein Diet and Resistance Training on Glucose Control and Inflammatory Profile of Visceral Adipose Tissue in Rats. Nutrients.

[37] Tibana R.A., Franco O.L., Cunha G.V., Sousa N.M.F., Neto I.V.S., Carvalho M.M., Almeida J.A., Durigan J.L.Q., Marqueti R.C., Navalta J.W. (2017). The Effects of Resistance Training Volume on Skeletal Muscle Proteome. Int. J. Exerc. Sci..

[38] De Sousa Neto I.V., Tibana R.A., da Silva L.G.O., de Lira E.M., do Prado G.P.G., de Almeida J.A., Franco O.L., Durigan J.L.Q., Adesida A.B., de Sousa M.V. (2020). Paternal Resistance Training Modulates Calcaneal Tendon Proteome in the Offspring Exposed to High-Fat Diet. Front. Cell Dev. Biol..

[39] De Sousa Neto I.V., Durigan J.L.Q., Guzzoni V., Tibana R.A., Prestes J., de Araujo H.S.S., Marqueti R.C. (2018). Effects of Resistance Training on Matrix Metalloproteinase Activity in Skeletal Muscles and Blood Circulation during Aging. Front. Physiol..

[40] Oliveira S.A., Pai-Silva M.D., Martinez P.F., Lima-Leopoldo A.P., Campos D.H., Leopoldo A.S., Politi O.M., Okoshi M.P., Okoshi K., Roberto P.C. (2010). Diet-induced obesity causes metabolic, endocrine and cardiac alterations in spontaneously hypertensive rats. Med. Sci. Monit..

[41] Carvalho M.R., Mendonça M.L.M., Oliveira J.M., Romanenghi R.B., Morais C.S., Ota G.E., Lima A.R., Oliveira R.J., Filiú W.F., Okoshi K. (2020). Influence of high-intensity interval training and intermittent fasting on myocardium apoptosis pathway and cardiac morphology of healthy rats. Life Sci..

[42] Basilio P.G., De Oliveira A.P.C., De Castro A.C.F., De Carvalho M.R., Zagatto A.M., Martinez P.F., Okoshi M.P., Okoshi K., Ota G.E., Dos Reis F.A. (2020). Intermittent fasting attenuates exercise training-induced cardiac remodeling. Arq. Bras. Cardiol..

[43] Martinez P.F., Bonomo C., Guizoni D.M., Oliveira-Junior S.A., Damatto R.L., Cezar M.D., Lima A.R., Pagan L.U., Seiva F., Bueno R.T. (2016). Modulation of MAPK and NF-κB Signaling Pathways by Antioxidant Therapy in Skeletal Muscle of Heart Failure Rats. Cell. Physiol. Biochem..

[44] Oliveira-Junior S.A., Martinez P.F., Guizoni D.M., Campos D.H.S., Fernandes T., Oliveira E.M., Okoshi M.P., Okoshi K., Padovani C.R., Cicogna A.C. (2014). AT1 Receptor Blockade Attenuates Insulin Resistance and Myocardial Remodeling in Rats with Diet-Induced Obesity. PLoS ONE.

[45] Bradford M.M. (1976). A Rapid and sensitive method for the quantitation of microgram quantities of protein utilizing the principle of protein-dye binding. Anal. Biochem..

[46] Martinez P.F., Okoshi K., Zornoff L.A.M., Carvalho R.F., Oliveira S.A., Lima A.R.R., Campos D.H.S., Damatto R.L., Padovani C.R., Nogueira C.R. (2010). Chronic heart failure-induced skeletal muscle atrophy, necrosis, and changes in myogenic regulatiory factors. Med. Sci. Monit..

[47] Lima A.R.R., Martinez P.F., Damatto R.L., Cezar M.D.M., Guizoni D.M., Bonomo C., Oliveira-Junior S.A., Silva M.D.-P., Zornoff L.A.M., Okoshi K. (2014). Heart Failure-Induced Diaphragm Myopathy. Cell. Physiol. Biochem..

[48] Aguiar A.F., De Souza R.W.A., Aguiar D.H., Aguiar R.C.M., Vechetti I.J., Dal-Pai-Silva M. (2011). Creatine does not promote hypertrophy in skeletal muscle in supplemented compared with nonsupplemented rats subjected to a similar workload. Nutr. Res..

[49] Damatto R., Martinez P., Lima A., Cezar M., Campos D., Junior S.O., Guizoni D., Bonomo C., Nakatani B., Silva M.D.P. (2013). Heart failure-induced skeletal myopathy in spontaneously hypertensive rats. Int. J. Cardiol..

[50] Pereira J.A.S.A., De Haan A., Wessels A., Moorman A.F., Sargeant A.J. (1995). The mATPase histochemical profile of rat type IIX fibres: Correlation with myosin heavy chain immunolabelling. Histochem. J..

[51] Qaisar R., Bhaskaran S., Van Remmen H. (2016). Muscle fiber type diversification during exercise and regeneration. Free. Radic. Biol. Med..

[52] Kraemer W.J., Ratamess N.A. (2004). Fundamentals of Resistance Training: Progression and Exercise Prescription. Med. Sci. Sports Exerc..

[53] Schoenfeld B.J., Grgic J., Ogborn D., Krieger J.W. (2017). Strength and Hypertrophy Adaptations Between Low- vs. High-Load Resistance Training: A Systematic Review and Meta-analysis. J. Strength Cond. Res..

[54] Carvalho L., Junior R.M., Barreira J., Schoenfeld B.J., Orazem J., Barroso R. (2022). Muscle hypertrophy and strength gains after resistance training with different volume-matched loads: A systematic review and meta-analysis. Appl. Physiol. Nutr. Metab..

[55] Škarabot J., Brownstein C.G., Casolo A., Del Vecchio A., Ansdell P. (2020). The knowns and unknowns of neural adaptations to resistance training. Eur. J. Appl. Physiol..

[56] Stotzer U.S., Pisani G.F.D., Canevazzi G.H.R., Shiguemoto G.E., Duarte A.C.G.D.O., Perez S.E.D.A., Selistre-De-Araújo H.S. (2018). Benefits of resistance training on body composition and glucose clearance are inhibited by long-term low carbohydrate diet in rats. PLoS ONE.

[57] Bae J.Y. (2020). Resistance Exercise Regulates Hepatic Lipolytic Factors as Effective as Aerobic Exercise in Obese Mice. Int. J. Environ. Res. Public Health.

[58] Folland J.P., Williams A.G. (2007). The adaptations to strength training morphological and neurological contributions to increased strength. Sport. Med..

[59] Wackerhage H., Schoenfeld B.J., Hamilton D., Lehti M., Hulmi J.J. (2019). Stimuli and sensors that initiate skeletal muscle hypertrophy following resistance exercise. J. Appl. Physiol..

[60] Ribeiro M.B.T., Guzzoni V., Hord J.M., Lopes G.N., Marqueti R.D.C., de Andrade R.V., Selistre-De-Araujo H.S., Durigan J.L.Q. (2017). Resistance training regulates gene expression of molecules associated with intramyocellular lipids, glucose signaling and fiber size in old rats. Sci. Rep..

[61] Deschenes M.R., Sherman E.G., Roby M.A., Glass E.K., Harris M.B. (2014). Effect of resistance training on neuromuscular junctions of young and aged muscles featuring different recruitment patterns. J. Neurosci. Res..

[62] Neto W.K., Gama E. (2017). Strength training and anabolic steroid do not affect muscle capillarization of middle-aged rats. Rev. Bras. Med. Esporte.

[63] Aagaard P., Andersen J.L., Dyhre-Poulsen P., Leffers A.-M., Wagner A., Magnusson S.P., Halkjaer-Kristensen J., Simonsen E.B. (2001). A mechanism for increased contractile strength of human pennate muscle in response to strength training: Changes in muscle architecture. J. Physiol..

[64] Kim J.-S., Park Y.-M., Lee S.-R., Masad I.S., Khamoui A.V., Jo E., Park B.-S., Arjmandi B.H., Panton L.B., Lee W.J. (2012). β-hydroxy-β-methylbutyrate did not enhance high intensity resistance training-induced improvements in myofiber dimensions and myogenic capacity in aged female rats. Mol. Cells.

[65] Douglas J., Pearson S., Ross A., McGuigan M. (2016). Chronic Adaptations to Eccentric Training: A Systematic Review. Sport. Med..

[66] Lourenço Í., Neto W.K., Amorim L.D.S.P., Ortiz V.M.M., Geraldo V.L., Ferreira G.H.D.S., Caperuto C., Gama E.F. (2020). Muscle hypertrophy and ladder-based resistance training for rodents: A systematic review and meta-analysis. Physiol. Rep..

[67] Braggion G.F., Ornelas E.D.M., Cury J.C.S., de Sousa J.P., Nucci R.A.B., Fonseca F.L.A., Maifrino L.B.M. (2020). Remodeling of the soleus muscle of ovariectomized old female rats submitted to resistance training and different diet intake. Acta Histochem..

[68] Csapo R., Gumpenberger M., Wessner B. (2020). Skeletal Muscle Extracellular Matrix—What Do We Know About Its Composition, Regulation, and Physiological Roles? A Narrative Review. Front. Physiol..

[69] McPherron A.C., Lee S.-J. (2002). Suppression of body fat accumulation in myostatin-deficient mice. J. Clin. Investig..

[70] Hamrick M.W., Pennington C., Webb C.N., Isales C.M. (2006). Resistance to body fat gain in ‘double-muscled’ mice fed a high-fat diet. Int. J. Obes..

[71] McPherron A.C., Lee S.-J. (1997). Double muscling in cattle due to mutations in the myostatin gene. Proc. Natl. Acad. Sci. USA.

[72] Grobet L., Martin L.J.R., Poncelet D., Pirottin D., Brouwers B., Riquet J., Schoeberlein A., Dunner S., Ménissier F., Massabanda J. (1997). A deletion in the bovine myostatin gene causes the double–muscled phenotype in cattle. Nat. Genet..

[73] Mosher D.S., Quignon P., Bustamante C.D., Sutter N.B., Mellersh C.S., Parker H.G., Ostrander E. (2007). A Mutation in the Myostatin Gene Increases Muscle Mass and Enhances Racing Performance in Heterozygote Dogs. PLoS Genet..

[74] Schuelke M., Wagner K.R., Stolz L.E., Hübner C., Riebel T., Kömen W., Braun T., Tobin J.F., Lee S.-J. (2004). Myostatin Mutation Associated with Gross Muscle Hypertrophy in a Child. N. Engl. J. Med..

[75] Raue U., Slivka D., Jemiolo B., Hollon C., Trappe S. (2006). Myogenic gene expression at rest and after a bout of resistance exercise in young (18–30 yr) and old (80–89 yr) women. J. Appl. Physiol..

[76] Hayashi S., Miyake M., Watanabe K., Aso H., Hayashi S., Ohwada S., Yamaguchi T. (2008). Myostatin preferentially down-regulates the expression of fast 2x myosin heavy chain in cattle. Proc. Jpn. Acad. Ser. B Phys. Biol. Sci..

[77] Santos A., Neves M., Gualano B., Laurentino G., Lancha A., Ugrinowitsch C., Lima F., Aoki M.S. (2014). Blood flow restricted resistance training attenuates myostatin gene expression in a patient with inclusion body myositis. Biol. Sport.

[78] Tang L., Luo K., Liu C., Wang X., Zhang D., Chi A., Zhang J., Sun L. (2014). Decrease in myostatin by ladder-climbing training is associated with insulin resistance in diet-induced obese rats. Chin. Med. J..

[79] Negaresh R., Ranjbar R., Baker J.S., Habibi A., Mokhtarzade M., Gharibvand M.M., Fokin A. (2019). Skeletal muscle hypertrophy, insulin-like growth factor 1, myostatin and follistatin in healthy and sarcopenic elderly men: The effect of whole-body resistance training. Int. J. Prev. Med..

[80] Deldicque L., Atherton P., Patel R., Theisen D., Nielens H., Rennie M.J., Francaux M. (2008). Effects of resistance exercise with and without creatine supplementation on gene expression and cell signaling in human skeletal muscle. J. Appl. Physiol..

[81] Volek J.S., Kraemer W.J., Bush J.A., Boetes M., Incledon T., Clark K.L., Lynch J.M. (1997). Creatine Supplementation Enhances Muscular Performance during High-Intensity Resistance Exercise. J. Am. Diet. Assoc..

[82] Cooke M.B., Brabham B., Buford T.W., Shelmadine B.D., McPheeters M., Hudson G.M., Stathis C., Greenwood M., Kreider R., Willoughby D.S. (2014). Creatine supplementation post-exercise does not enhance training-induced adaptations in middle to older aged males. Eur. J. Appl. Physiol..

[83] Sun M., Jiao H., Wang X., Li H., Zhou Y., Zhao J., Lin H. (2022). The regulating pathway of creatine on muscular protein metabolism depends on the energy state. Am. J. Physiol. Physiol..

[84] Mazzetti S., Douglass M., Yocum A., Harber M. (2007). Effect of Explosive versus Slow Contractions and Exercise Intensity on Energy Expenditure. Med. Sci. Sport. Exerc..

